# A Real-World Perspective of Co-amoxiclav Prescription Pattern With Probiotics for Pediatric Patients With Respiratory Tract Infections: Results of Quantitative and Qualitative Approach in Indian Outpatient Settings

**DOI:** 10.7759/cureus.36269

**Published:** 2023-03-16

**Authors:** Jaydeep Choudhury, Ashish Makkar, Vipul Sharma, S P Karamath, Vishal Parmar, Paras Kumar J, Krishna C Veligandla, Colette S Pinto, Amey Mane, Rahul Rathod, Bhavesh P Kotak

**Affiliations:** 1 Department of Pediatrics, Institute of Child Health, Kolkata, IND; 2 Department of Pediatrics, Makkar Multispeciality Hospital, Delhi, IND; 3 Department of Pediatrics, Prakash Maternity and Child Clinic, Gurugram, IND; 4 Department of Pediatrics, Tan Man Child Care Clinic, Chennai, IND; 5 Department of Pediatrics, Bangalore Super Speciality Centre, Bengaluru, IND; 6 Medical Affairs, Dr. Reddy’s Laboratories Ltd., Hyderabad, IND; 7 Clinical Research, Medical Affairs, Dr. Reddy’s Laboratories Ltd., Hyderabad, IND; 8 Ideation and Clinical Research, Medical Affairs, Dr. Reddy’s Laboratories Ltd., Hyderabad, IND

**Keywords:** respiratory tract infections, probiotics, co-amoxiclav, antibiotics, antibiotic-associated diarrhea

## Abstract

Background: Probiotics are co-prescribed with co-amoxiclav to prevent antibiotic-associated diarrhea (AAD). The study assesses the co-prescription pattern of probiotics with co-amoxiclav in pediatric patients with respiratory tract infections (RTIs).

Methods: This was a mixed methods research study with a retrospective study and a prospective survey. The retrospective part included a multicenter, observational, real-world study utilizing patients’ electronic medical records for three years (2018-2020) from seven outpatient pediatric clinics and hospitals. The qualitative evaluation was performed with a predefined questionnaire.

Results: The patients having RTIs (N=984) were prescribed Clamp^® ^(46.7%), CAA (23.8%), and CAM (29.5%). The mean age of the patients was 4.05 years, with 59.25% males and most patients having upper RTIs. Co-amoxiclav was prescribed twice daily for one to 15 days. A significantly lesser number of probiotic co-prescriptions were observed with Clamp^®^ (19.57%) than with CAA (38.46%) and CAM (29.31%) at baseline (*p*<0.001). Similar findings were observed for follow-up visits one and two. *Saccharomyces* *boulardii*, *Bacillus clausii*,and lactic acid bacillus were the most commonly co-prescribed probiotics. The qualitative evaluation indicated that most clinicians were aware of the co-amoxiclav-related gastrointestinal side effects and the benefits of probiotics in preventing them.

Conclusion: The frequency of co-prescriptions of probiotics with Clamp^®^ among pediatric patients with RTIs was significantly less, potentially indicating better gastrointestinal tolerability.

## Introduction

Respiratory tract infections (RTIs) present a significant challenge to the health system, particularly in developing countries, and are the leading cause of mortality and morbidity among children under five years [1]. The National Family Health Survey (NFHS-5) conducted during 2019-2021 reported a 2.8% prevalence of acute respiratory infection (ARI) among children under the age of five years in India [2]. Additionally, a systematic analysis conducted to identify the cause of mortality in children under five years in India (2000-15) reported that pneumonia, one of the major manifestations of lower respiratory infections (LRI), is the second leading cause of mortality, and contributed to 15.9% deaths [3].

Amoxicillin/clavulanate or co-amoxiclav is a broad-spectrum antibacterial that has been clinically used in a wide range of indications for over 20 years and is predominantly used to treat community-acquired RTIs [4]. Co-amoxiclav was developed as a result of increasing resistance to amoxicillin due to the beta-lactamase-producing strains of various bacteria, which was resolved by adding a beta-lactamase inhibitor, i.e., clavulanic acid [5]. The production method of amoxicillin impacts the quality of the resulting antibiotic formulation. As per good manufacturing practices (GMPs), it is essential that production activities for amoxicillin be carried out in a dedicated facility with the appropriate containment strategy and controls to avoid cross-contamination since it can cause an anaphylaxis reaction [6]. A biocatalytic or enzymatic synthesis method should be used over the traditional chemical method owing to the lower cost of raw materials, fewer steps, use of ambient temperature, pressure and pH, high product quality, decrease or elimination of waste production, and no requirement of toxic or hazardous reagents or solvents [7].

Co-amoxiclav is effective for a variety of pediatric infectious diseases, including acute otitis media (AOM), sinusitis, pneumonia, urinary tract infections (UTIs), and infections of the skin and soft tissues [8]. The Centers for Disease Control and Prevention (CDC) described in their pediatric outpatient treatment recommendations that co-amoxiclav is recommended for RTIs, including acute sinusitis and acute otitis media (AOM) [9]. National Treatment Guidelines for Antimicrobial Use in Infectious Diseases 2016 recommend co-amoxiclav for AOM (severe cases), acute sinusitis with URTIs, and uncomplicated UTIs [10].

Antibiotic-associated diarrhea (AAD) is the primary complication reported in patients prescribed antibiotics. Gut dysbiosis and depletion of beneficial commensal bacteria, including *Bifidobacterium* and *Lactobacillus, *have been reported with the administration of beta-lactam antibiotics. Additionally, antibiotic treatment disrupts the different components of the intestinal barrier [11]. Probiotics, when taken with antibiotics, are known to reduce the risk of AAD in children [12]. They are live microorganisms that, when administered adequately, confer health benefits on the host. Probiotics help improve gut microbial composition, competing with pathogens for nutrients, growth, and adhesion, strengthening the gut mucosal barrier, resulting in an anti-inflammatory effect and modulation of the immune system [13]. Further, two groups of Asian experts have proposed that probiotics can be considered for preventing AAD [14,15]. A systematic review and meta-analysis by Blaabjerg et al. (2017) also highlighted the beneficial use of probiotics in preventing AAD among outpatients [16].

The justification for co-administration of probiotics with antibiotics might vary depending on the patient profile (such as recurrent infections, etc.) and the manufacturing quality of the antibiotic formulations. Thus, it can be hypothesised that the need for a physician to prescribe probiotics during the follow-up visits can be attributed to the antibiotics-associated side effects. Therefore, a real-world evidence-based study using retrospective electronic medical records (EMRs) database was planned to evaluate the co-prescription pattern of probiotics with co-amoxiclav among the pediatric population at baseline and follow-up visits. Additionally, a qualitative evaluation was conducted to determine clinicians' opinions on co-amoxiclav and adding probiotics to co-amoxiclav to provide insights into their current practices and prescribing decisions.

## Materials and methods

A mixed methods research, including a retrospective multicenter quantitative study and a prospective qualitative survey, was conducted.

Quantitative approach

Study Design and Data Sources

A multicenter, retrospective, observational, real-world study was conducted to determine the co-prescription pattern of probiotics prescribed with co-amoxiclav. EMRs of patients meeting the inclusion criteria were collected for three years (2018-2020) from seven outpatient pediatric clinics and hospitals’ outpatient departments (OPDs) across India. Patients' data were retrieved for baseline, first follow-up (one to 15 days after baseline), and second follow-up visits (two to seven days after the first follow-up visit). The follow-up durations were selected based on the real-world practice of clinicians while prescribing co-amoxiclav for RTIs in pediatric patients. The anonymized and de-identified data were analyzed to maintain patients' confidentiality.

Study Population

The study included pediatric patients ≤12 years of age with a confirmed clinical diagnosis of respiratory diseases, including URTIs [cough and cold/ARI, AOM, pharyngitis, and tonsillitis] and LRTI [unspecified LRI, wheezing-associated respiratory illness (WARI), bronchitis, bronchopneumonia, and reactive airway disease], and treated with co-amoxiclav (tablet, oral suspension, and syrup). Additionally, all the patients with different comorbidities were included in the study to represent a real-world scenario. Patients with incomplete medical records were excluded from the study. The patients were stratified into three groups based on the study drugs, i.e., patients prescribed with Clamp® (228.5 mg, 457 mg) in Group I, patients prescribed with co-amoxiclav brand CAA (228.5 mg, 457 mg, 625 mg) in Group II, and patients prescribed with co-amoxiclav brand CAM (156.25 mg, 228.5 mg, 375 mg, 457 mg) in Group III.

Study Variables and Outcomes

Patient data from EMRs, including demographic characteristics, chief complaints, drug treatment details, and concomitant medications, were assessed. The prescription patterns of probiotics and co-amoxiclav were evaluated at baseline and two follow-up visits. Safety was analyzed by determining the proportion of patients reporting adverse events following treatment initiation with co-amoxiclav.

Qualitative approach

For the qualitative/prospective approach, a self-administered predefined questionnaire encompassing open and close-ended questions was provided to the clinicians to record their responses. The paper-based questionnaire was created by the principal investigator and was designed to meet the study objectives. The clinicians' considerations for antibiotic usage, especially co-amoxiclav and adding probiotics to co-amoxiclav, were evaluated. The questionnaire was provided to 48 clinicians practicing at pediatric clinics and hospitals.

Ethical Statement

The study was conducted as per the protocol and principles of the Declaration of Helsinki. Ethical approval was obtained from the Royal Pune Independent Ethics Committee (RPIEC), Pune, India (RPIEC200422; dated: 12th April 2022).

Statistical Analysis

Data were analyzed using R Studio 3.2.1 (RStudio Corporation, Boston, MA, USA). Study data were presented using descriptive statistics. Continuous variables were presented as mean and median (range). Categorical variables were presented as frequency and percentages/proportions. Mann-Whitney U test was used to evaluate the age difference, and Chi-Square Test was used to assess the gender difference across the groups. Statistical significance was considered at p<0.05. For qualitative analysis, themes were generated from the clinician's responses, and the perspectives/responses were presented using descriptive statistics (frequency and percentages/proportions).

## Results

Quantitative approach

A total of 984 patients having RTIs were included in the analysis, of which 46.7% (460) patients were prescribed Clamp® (Group I), 23.8% (234) were prescribed CAA (Group II), and 29.5% (290) were prescribed CAM (Group III). The mean age of the patients in Groups I, II, and III were 3.71, 4.45, and 4.26 years, respectively. A predominance of male patients (59.25%) was observed in the study population (Table 1).

**Table 1:** Baseline characteristics of patients with respiratory tract infections (RTIs) (N=984)

Parameters	Total	Group I (Clamp®)	Group II (CAA)	Group III (CAM)
Number of patients	984	460	234	290
Age (Years)				
Mean	4.05	3.71	4.45	4.26
Median (Range)	4 (0-12)	3 (0-12)	4 (0-12)	4 (0-12)
*p*-value (Intergroup comparison)	-	0.001		
Gender; n (%)				
Female	397 (40.35%)	212 (46.09%)	82 (35.04%)	103 (35.52%)
Male	583 (59.25%)	244 (53.04%)	152 (64.96%)	187 (64.48%)
Data not available	4 (0.41%)	4 (0.87%)	0 (0%)	0 (0%)
*p*-value (Intergroup comparison)	-	0.002		

Among children with URTIs, cold and cough/ARI was the most common condition reported by 32.11% (316) patients, followed by AOM, pharyngitis, and tonsillitis in 7.22% (71), 4.27% (42), and 1.32% (13) patients, respectively. Patients with URTIs mostly complained of cough, fever, cold/running nose, irritability/excessive crying, and ear pain. Further, unspecified LRTI was the most frequent condition reported by 11.08% (109) patients, followed by WARI in 1.93% (19), bronchitis in 1.22% (12), bronchopneumonia in 0.91% (nine), and reactive airway disease in 0.51% (five) patients. Patients with LRTIs mostly complained of cough/wheeze, fever, cold/running nose, and throat congestion (Table 2).

**Table 2:** Chief complaints of patients with respiratory tract infections (RTIs) (N=984) *Other complaints (120): Throat congestion (52); Vomiting (13); Throat pain (10); Headache (3); Loose stool (3); Sore throat (2); Ear pain (2); Constipation (2); Tonsils enlarged (2); Ear itching (2); Enlarged lymph node (2); Mild throat hyperemic (2); Stomach pain (2); Mouth ulcer (2); Tight motion (2); Abdominal pain (1); Boil over buttock (1); Breathlessness (1); Chest crept (1); Dysuria (1); Ear tugging (1); Face rashes (1); Face swelling (1); Irritability (1); Low appetite (1); Mild dehydration (1); Muscular pain (1); Neck swelling (1); Night cry (1); Oral ulcer (1); Rhinorrhea (1); Rhonchi (1); Throat ulcer (1); Wheezing (1) **Other complaints (26): Cough (12); Ear discharge/ Ear wax (6); Cold/Running nose (5); Ear canal swelling (1); Redness (1); Headache (1) ***Other complaints (19): Throat congestion (6); Throat pain (6); Throat swelling (1); Ear pain (1); Abdominal pain (1); Vomiting (1); Itching (1); Skin infection (1); Rash (1) ****Other complaints (14): Cold/Running nose (4); Enlarged tonsils/swelling (2); Throat pain (2); Rash (2); Vomiting (1); Headache (1); Abdominal pain (1); Bilateral jugulodigastric (1) #Other complaints (21): Vomiting (7); Throat congestion (5); Body ache (1); Ear pain (1); Enlarged lymph node (1); Excessive crying (1); Eye pain (1); Leg pain (1); Mild dehydration (1); Nasal congestion (1); Squint (1) ##Other complaints (8): Throat congestion (2); Vomiting (1); Loss of Appetite (1); Chest crept (1); Rhonchi (1); Tachypnoea (1); Loose stools (1) ###Other complaints (4): Vomiting (1); Skin rashes (1); Itching (1); Throat congestion (1) ####Other complaints (15): Chest crept (4); Loose stools (4); Rhonchi (3); Cold (2); Breathlessness/Dyspnoea/ Tachypnoea (2) #####Other complaint (1): Respiratory distress (1)

Parameters	Total	Group I (Clamp®)	Group II (CAA)	Group III (CAM)
Number of patients	984	460	234	290
Chief complaints				
Upper respiratory tract infections (URTI)				
Cough and cold/Acute respiratory infection (ARI)	316 (32.11%)	158 (34.35%)	57 (24.36%)	101 (34.83%)
Cough	220 (69.62%)	128 (81.01%)	23 (40.35%)	69 (68.32%)
Fever/Febrile	206 (65.19%)	125 (79.11%)	22 (38.6%)	59 (58.42%)
Cold/Running nose	199 (62.97%)	120 (75.95%)	20 (35.09%)	59 (58.42%)
Other complaints (120)*				
Acute otitis media (AOM)	71 (7.22%)	42 (9.13%)	14 (5.98%)	15 (5.17%)
Fever	44 (61.97%)	29 (69.05%)	6 (42.86%)	9 (60%)
Irritability/excessive crying	39 (54.93%)	25 (59.52%)	6 (42.86%)	8 (53.33%)
Ear pain	30 (42.25%)	17 (40.48%)	5 (35.71%)	8 (53.33%)
Other complaints (26)**				
Pharyngitis	42 (4.27%)	18 (3.91%)	19 (8.12%)	5 (1.72%)
Fever/Febrile	17 (40.48%)	12 (66.67%)	3 (15.79%)	2 (40%)
Cold/Running nose	15 (35.71%)	9 (50%)	4 (21.05%)	2 (40%)
Cough	12 (28.57%)	7 (38.89%)	3 (15.79%)	2 (40%)
Other complaints (19)***				
Tonsillitis	13 (1.32%)	5 (1.09%)	1 (0.43%)	7 (2.41%)
Fever/Febrile	9 (69.23%)	4 (80%)	1 (100%)	4 (57.14%)
Cough	7 (53.85%)	3 (60%)	0 (0%)	4 (57.14%)
Throat congestion	7 (53.85%)	3 (60%)	1 (100%)	3 (42.86%)
Other complaints (14)****				
Lower respiratory tract infections (LRTI)				
Unspecified lower respiratory infection (LRI)	109 (11.08%)	77 (16.74%)	23 (9.83%)	9 (3.1%)
Cough/Wheeze	84 (77.06%)	68 (88.31%)	13 (56.52%)	3 (33.33%)
Fever	64 (58.72%)	55 (71.43%)	7 (30.43%)	2 (22.22%)
Cold/Running nose	62 (56.88%)	57 (74.03%)	2 (8.7%)	3 (33.33%)
Other complaints (21)^#^				
Wheezing-associated respiratory illness (WARI)	19 (1.93%)	12 (2.61%)	4 (1.71%)	3 (1.03%)
Cough/Wheeze	15 (78.95%)	12 (100%)	1 (25%)	2 (66.67%)
Cold/Running nose	11 (57.89%)	10 (83.33%)	0 (0%)	1 (33.33%)
Fever/Febrile	8 (42.11%)	7 (58.33%)	0 (0%)	1 (33.33%)
Other complaints (8)^##^				
Bronchitis	12 (1.22%)	12 (2.61%)	0 (0%)	0 (0%)
Fever/Febrile	6 (50%)	6 (50%)	0 (0%)	0 (0%)
Cough/Wheeze	8 (66.67%)	8 (66.67%)	0 (0%)	0 (0%)
Cold	9 (75%)	9 (75%)	0 (0%)	0 (0%)
Other complaints (4)^###^				
Bronchopneumonia	9 (0.91%)	2 (0.43%)	1 (0.43%)	6 (2.07%)
Fever/Febrile	4 (44.44%)	1 (50%)	0 (0%)	3 (50%)
Cough	4 (44.44%)	1 (50%)	0 (0%)	3 (50%)
Throat congestion	3 (33.33%)	1 (50%)	0 (0%)	2 (33.33%)
Other complaints (15)^####^				
Reactive airway disease	5 (0.51%)	3 (0.65%)	0 (0%)	2 (0.69%)
Fever/Febrile	2 (40%)	2 (66.67%)	0 (0%)	0 (0%)
Cough/Wheeze	5 (100%)	3 (100%)	0 (0%)	2 (100%)
Cold	2 (40%)	2 (66.67%)	0 (0%)	0 (0%)
Other complaint (1)^#####^				

According to the drug treatment details, at the baseline visit, Clamp® (228.5 mg and 457 mg), CAA (228.5 mg, 457 mg, and 625 mg), and CAM (156.25 mg, 228.5 mg, 375 mg, and 457 mg) were prescribed in Groups I, II, and III, respectively. All three co-amoxiclav brands were prescribed twice daily for an average duration of 5.32 days. At follow-up visits one and two, only two doses of co-amoxiclav, i.e., 228.5 mg and 457 mg, were prescribed twice daily. The average duration of treatment was 4.71 days at follow-up visit one, ranging from one to 15 days in Group I, four to seven days in Group II, and four to 14 days in Group III, respectively. At follow-up visit two, the average duration of treatment was 3.18 days, ranging from two to five days in all three groups (Table 3).

**Table 3:** Details of co-amoxiclav prescribed to patients with respiratory tract infections (RTIs) (N=984)

Drug details	Total	Group I (Clamp®)	Group II (CAA)	Group III (CAM)
Number of patients	984	460	234	290
Baseline visit				
Dose of drug (mg)				
156.25 mg	1	-	-	1
228.5 mg	387	157	89	141
375 mg	1	-	-	1
457 mg	333	175	53	105
625 mg	2	-	2	-
Data not available	260	128	90	42
Frequency; n (%)				
OD	-	-	-	-
BD	466	170	138	158
Data not available	518	290	96	132
Duration (Days)				
Average	5.32	5.33	5.76	5.37
Range	3-9	3-9	3-9	3-9
Follow-up visit 1 (1-15 Days)				
Dose of drug (mg)				
228.5 mg	340	145	88	107
457 mg	163	52	50	61
Data not available	481	263	96	122
Frequency; n (%)				
OD	-	-	-	-
BD	466	170	138	158
Data not available	518	290	96	132
Duration (Days)				
Average	4.71	4.72	4.65	4.76
Range	1-15	1-15	4-7	4-14
Follow-up visit 2 (2-7 Days)				
Dose of drug (mg)				
228.5 mg	335	144	88	103
457 mg	135	27	52	56
Data not available	514	289	94	131
Frequency; n (%)				
OD	-	-	-	-
BD	466	170	138	158
Data not available	518	290	96	132
Duration (Days)				
Average	3.18	2.67	2.69	3.35
Range	2-5	2-5	2-5	2-5

The patients took various concomitant drugs (except probiotics) during the treatment. The most commonly prescribed concomitant medications in Group I, II, and III included paracetamol, cetirizine + ambroxol combination, ambroxol + levosalbutamol + guaifenesin combination, and ibuprofen + paracetamol combination (Table 4).

**Table 4:** Concomitant medication drugs prescribed to patients with respiratory tract infections (RTIs) (N=984)

Groups	Concomitant medications	Dose	Frequency	Count	Duration (Days)	
					Mean	Range
Group I (Clamp^®^)	Cetirizine + Ambroxol	5 ml	BD	2	7	7
	Ambroxol + Levosalbutamol + Guaifenesin	3 ml/ [(7.5 mg/ml) + (0.25 mg/ml) + (12.5 mg/ml)]	TDS	1	5	5
		3 ml	TDS	6	5	5
	Paracetamol	125 mg	BD	31	NA	NA
		150 mg	BD	2	3	3
		250 mg	BD	26	3	3
	Ibuprofen	100 mg/5 mL	BD	2	3	3
		150 mg/7.5 ml	BD	1	3	3
	Ibuprofen + Paracetamol	10 mg/kg	BD	3	5	5
		150 mg	BD	5	5	5
		200 mg	BD	2	5	5
		250 mg	BD	2	5	5
	Chlorpheniramine maleate + Phenylephrine	5 ml	TDS	1	2	2
	Cetirizine + Ambroxol	5 ml	TDS	36	5	5
	Phenylephrine + Chlorpheniramine maleate + Dextromethorphan hydrobromide	10 ml	TDS	3	5	5
	Ambroxol + Guaifenesin + Terbutaline	3 ml/ [ (15 mg/5 ml) + (50 mg/5ml) + (1.25 mg/5 ml)]	TDS	1	7	7
		3 ml	TDS	1	7	7
		5 ml	TDS	5	7	7
Group II (CAA)	Cetirizine + Ambroxol	5 ml	BD	3	7	7
	Ambroxol + Levosalbutamol + Guaifenesin	3 ml/ [(7.5 mg/ml) + (0.25 mg/ml) + (12.5 mg/ml)]	TDS	1	5	5
		125 mg	BD	16	3	3
		250 mg	BD	53	3	3
	Ibuprofen	100 mg/5 mL	BD	1	3	3
	Ibuprofen + Paracetamol	10 mg/kg	BD	1	5	5
		150 mg	BD	4	5	5
		200 mg	BD	1	5	5
		250 mg	BD	1	5	5
	Cetirizine + Ambroxol	5 ml	TDS	14	5	5
	Phenylephrine + Chlorpheniramine maleate + Dextromethorphan hydrobromide	10 ml/ [(5 mg) + (2 mg) + (10 mg)]	TDS	1	7	7
		10 ml	TDS	5	5	5
Group III (CAM)	Cetirizine + Ambroxol	5 ml	BD	1	7	7
	Ambroxol + Levosalbutamol + Guaifenesin	3 ml/ [(7.5 mg/ml) + (0.25 mg/ml) + (12.5 mg/ml)]	TDS	2	5	5
		3 ml	TDS	1	5	5
		125 mg	BD	15	3	3
		150 mg	BD	1	3	3
		250 mg	BD	68	3	3
	Ibuprofen	100 mg/5 mL	BD	3	3	3
	Ibuprofen + Paracetamol	150 mg	BD	5	5	5
		200 mg	BD	2	5	5
	Cetirizine + Ambroxol	5 ml	TDS	7	5	5
	Phenylephrine + Chlorpheniramine maleate + Dextromethorphan hydrobromide	10 ml	TDS	1	5	5
	Ambroxol + Guaifenesin + Terbutaline	5 ml	TDS	1	7	7

The prescription patterns of probiotics with co-amoxiclav were evaluated by assessing the number of patients prescribed probiotics at baseline and follow-up visits. At baseline, a significantly lesser number of probiotic co-prescriptions were observed in Group I [19.57% (90)], as compared to Groups II [38.46% (90)] and III [29.31% (85)] (*p*<0.001). A similar pattern of probiotic co-prescription was observed during the follow-up visits, with lesser probiotic co-prescription in Group I as compared to Groups II and III at follow-up visit one [22.39% (103) vs. 32.48% (76) vs. 26.21% (76); p=0.016] and visit two [16.3% (75) vs. 28.21% (66) vs. 22.76% (66); p=0.001], respectively.

The probiotics co-prescribed with co-amoxiclav in Groups I, II, and III, respectively, are presented in Table 5. Overall, *Saccharomyces boulardii* (*S. boulardii*) was the most prescribed probiotic in 64 and 59 patients at baseline and follow-up visit one, followed by *Bacillus clausii* (*B. clausii*) in 60 and 58 patients and lactic acid bacteria (LAB) in 29 and 28 patients, respectively. At a follow-up visit two, *B. clausii* and *S. boulardii* prescriptions were almost equal at 58 vs. 57, while LAB was prescribed in 31 patients. In Group I, at all visits, patients were primarily co-prescribed with probiotics, including *B. clausii*, followed by *S. boulardii*, and LAB. In Groups II and III, S. boulardii, B. clausii, and LAB were the most co-prescribed probiotics at all visits. Other frequently co-prescribed probiotics included LAB + niacinamide + pyridoxine, LAB + niacinamide + folic acid + pyridoxine + zinc, and *Streptococcus faecalis* + *Clostridium butyricum* + *B. mesentericus* + LAB.

**Table 5:** Prescription patterns of probiotics along with co-amoxiclav in patients with respiratory tract infections (RTIs) (N=984) *Other probiotics: (Streptococcus faecalis + Clostridium butyricum + B. mesentericus + LAB),  (LAB + Niacinamide + Folic acid + Pyridoxine + Zinc), B. subtilis,  Lactobacillus rhamnosus GG **Other probiotics: (Streptococcus faecalis + Clostridium butyricum + B. mesentericus + LAB), (LAB + Niacinamide + Folic acid + Pyridoxine + Zinc), (Lactobacillus acidophilus + Lactobacillus casei + Colostrum + Vitamin C + Zinc), (LAB + Niacinamide + Pyridoxine),  Lactobacillus rhamnosus GG ***Other probiotics: (Streptococcus faecalis + Clostridium butyricum + B. mesentericus + LAB), (LAB + Niacinamide + Folic acid + Pyridoxine + Zinc), B. subtilis, (LAB + Niacinamide + Folic acid + Pyridoxine + Zinc) #Other probiotics: (Streptococcus faecalis + Clostridium butyricum + B. mesentericus + LAB), B. subtilis, (LAB + Niacinamide + Folic acid + Pyridoxine + Zinc), (Lactobacillus acidophilus + Lactobacillus casei + Colostrum + Vitamin C + Zinc), Lactobacillus rhamnosus GG ##Other probiotics: (Streptococcus faecalis + Clostridium butyricum + B. mesentericus + LAB), (Lactobacillus acidophilus + Lactobacillus casei + Colostrum + Vitamin C + Zinc), Lactobacillus rhamnosus GG ###Other probiotics: (LAB + Niacinamide + Folic acid + Pyridoxine + Zinc), B. subtilis, (Streptococcus faecalis + Clostridium butyricum + B. mesentericus + LAB) †Other probiotics: (LAB + Niacinamide + Folic acid + Pyridoxine + Zinc), B. subtilis, Lactobacillus rhamnosus GG ††Other probiotics: Lactobacillus rhamnosus GG, (Streptococcus faecalis + Clostridium butyricum + B. mesentericus + LAB) †††Other probiotics: (LAB + Niacinamide + Folic acid + Pyridoxine + Zinc), B. subtilis, (Lactobacillus acidophilus + Lactobacillus casei + Colostrum + Vitamin C + Zinc), (Streptococcus faecalis + Clostridium butyricum + B. mesentericus + LAB)

Groups	Probiotics	Formulation	Dose	Frequency	N	Data not available	Duration (Days)	
							Mean	Range
	Baseline visit							
Group I (Clamp®)	S. boulardii	Sachet	250 mg	BD	16	3	5.38	5-7
	Lactic acid bacillus (LAB) + Niacinamide + Pyridoxine	Syrup	5 ml	BD	5	0	5.40	5-7
	*B. clausii* Brand 1	Suspension	5 ml	BD	19	0	6.05	5-7
	*B. clausii *Brand 2	Suspension	5 ml	OD	7	0	5.29	5-7
	LAB	Syrup	5 ml	OD	9	0	5	5
	Other probiotics*							
Group II (CAA)	S. boulardii	Sachet	250 mg	BD	24	1	5.50	5-7
	*B. clausii* Brand 1	Suspension	5 ml	BD	25	0	6.44	5-7
	*B. clausii *Brand 2	Suspension	5 ml	OD	3	0	6.33	5-7
	*B. clausii *Brand 3	Suspension	5 ml	OD	4	0	6.00	5-7
	LAB	Syrup	5 ml	OD	8	0	5.00	5
	Other probiotics**							
Group III (CAM)	S. boulardii	Sachet	250 mg	BD	24	5	5.50	5-7
	*B. clausii* Brand 1	Suspension	5 ml	BD	16	0	6.75	5-7
	*B. clausii* Brand 3	Suspension	5 ml	OD	6	0	6.33	5-7
	LAB + Niacinamide + Pyridoxine	Syrup	5 ml	BD	3	0	5.00	5
	LAB	Syrup	5 ml	OD	12	0	5.00	5
	Other probiotics***							
	Follow-up visit 1 (1-15 Days)							
Group I (Clamp®)	S. boulardii	Sachet	250 mg	BD	14	0	4.71	4-5
	LAB + Niacinamide + Pyridoxine	Syrup	5 ml	BD	5	3	4.8	4-5
	*B. clausii* Brand 1	Suspension	5 ml	BD	21	0	4.71	4-5
	*B. clausii* Brand 2	Suspension	5 ml	OD	7	0	4.43	4-5
	LAB	Syrup	5 ml	OD	8	0	4.5	4-5
	Other probiotics^#^							
Group II (CAA)	S. boulardii	Sachet	250 mg	BD	25	0	4.76	4-7
	*B. clausii *Brand 3	Suspension	5 ml	OD	4	0	4.75	4-5
	*B. clausii *Brand 1	Suspension	5 ml	BD	20	0	5.00	3-7
	(LAB + Niacinamide + Folic acid + Pyridoxine + Zinc)	Syrup	5 ml	BD	6	0	3.00	3
	LAB	Syrup	5 ml	OD	8	0	4.75	4-5
	Other probiotics^##^							
Group III (CAM)	S. boulardii	Sachet	250 mg	BD	20	0	4.75	4-5
	*B. clausii *Brand 1	Suspension	5 ml	BD	17	0	5.24	4-7
	*B. clausii *Brand 3	Suspension	5 ml	OD	6	0	4.50	4-5
	LAB + Niacinamide + Pyridoxine	Syrup	5 ml	BD	3	0	4.67	4-5
	LAB	Syrup	5 ml	OD	12	0	4.50	3-5
	Other probiotics^###^							
	Follow-up visit 2 (2-7 Days)							
Group I (Clamp®)	S. boulardii	Sachet	250 mg	BD	13	2	2	2
	LAB + Niacinamide + Pyridoxine	Syrup	5 ml	BD	5	0	2	2
	*B. clausii* Brand 1	Suspension	5 ml	BD	21	0	2	2
	*B. clausii* Brand 2	Suspension	5 ml	OD	7	0	2	2
	LAB	Syrup	5 ml	OD	11	0	2.18	2-4
	Other probiotics^†^							
Group II (CAA)	S. boulardii	Sachet	250 mg	BD	24	0	2.00	2 Days
	*B. clausii *Brand 3	Suspension	5 ml	OD	4	0	2.00	2 Days
	(LAB + Niacinamide + Folic acid + Pyridoxine + Zinc)	Syrup	5 ml	BD	3	0	2.00	2 Days
	*B. clausii *Brand 1	Suspension	5 ml	BD	20	0	2.00	2 Days
	LAB	Syrup	5 ml	OD	8	0	2.00	3 Days
	Other probiotics^††^							
Group III (CAM)	S. boulardii	Sachet	250 mg	BD	20	0	2.95	2-5 Days
	*B. clausii *Brand 3	Suspension	5 ml	OD	6	0	2.00	2 Days
	LAB + Niacinamide + Pyridoxine	Syrup	5 ml	BD	3	0	2.00	2 Days
	*B. clausii* Brand 1	Suspension	5 ml	BD	17	0	2.59	2-7 Days
	LAB	Syrup	5 ml	OD	12	0	2.50	2-5 Days
	Other probiotics^†††^							

Qualitative approach

**Figure 1:**
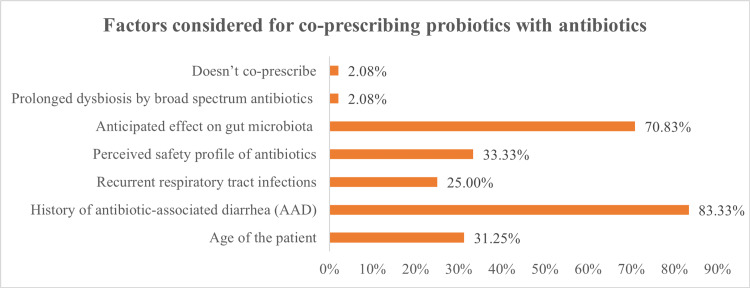
Factors considered for co-prescribing probiotics with antibiotics (N=48)

**Figure 2:**
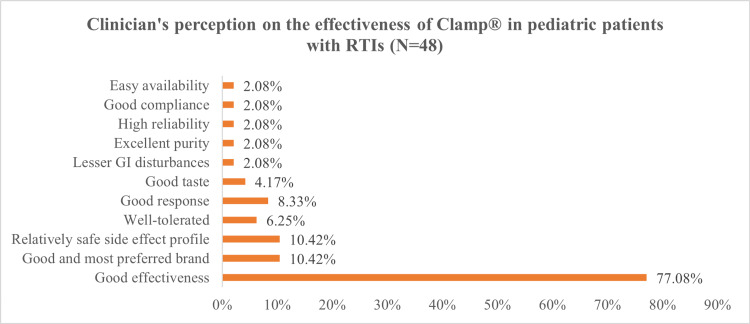
Clinician's perception of the effectiveness profile of Clamp® in pediatric patients with respiratory tract infections (RTIs) (N=48)

**Figure 3:**
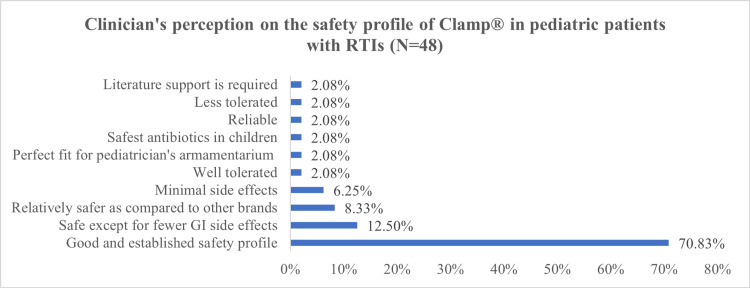
Clinician's perception of the safety profile of Clamp® in pediatric patients with respiratory tract infections (RTIs) (N=48)

The clinicians' considerations (N=48) for choosing co-amoxiclav and adding probiotics to co-amoxiclav were assessed using a self-administered predefined questionnaire (Table 6 and Figures 1-3). Most clinicians were pediatricians [40 (83.33%)], followed by general physicians [5 (10.42%)], and consulting physicians [3 (6.25%)]. The clinicians were practicing in tier 1 [36 (75%)] and tier 2 cities [12 (25%)], and the majority had more than 10 years of practice. According to 27.08% of clinicians, the recommended duration of co-amoxiclav treatment therapy for various indications in children is five to seven days. Nearly 92% of clinicians agreed that non-adherence to oral antibiotic treatment in children poses a challenge, the primary key consequences of which can be antimicrobial resistance (59.09%), recurrence of infection (11.36%), and disease progression and complications (9.09%). Further, according to 85.42% of clinicians, challenges with non-compliance to antibiotic therapy could be addressed using a 60 ml dosage of co-amoxiclav (instead of the current 30 ml dosage). Of these, 78.05% advised it for RTIs, with 46.88% of clinicians recommending it for URTIs and 37.50% for LRTIs. The patient considerations while prescribing the 60 ml dosage included the weight of children (17.07%), older children who are uncomfortable taking tablets (12.2%), children requiring a longer duration of the course (7.32%), all children (4.88%), and children with disease progression and complications (2.44%).

**Table 6:** Clinicians' preference assessment (N=48)

Clinicians’ Responses	n (%)
1. Advised duration of co-amoxiclav treatment therapy for various indications in children (days)	
5	9 (18.75%)
5 – 7	13 (27.08%)
5 – 10	4 (8.33%)
5 – 14	5 (10.42%)
6 – 10	1 (2.08%)
6 – 14	1 (2.08%)
7 – 10	9 (18.75%)
7 – 14	5 (10.42%)
10	1 (2.08%)
2. Non-adherence to oral antibiotic treatment in children poses a challenge	
Yes	44 (91.67%)
No	4 (8.33%)
If yes, the key consequences	
Antimicrobial resistance	26 (59.09%)
Recurrence of infection	5 (11.36%)
Disease progression and complications	4 (9.09%)
Worsening of illness leading to hospitalization	3 (6.82%)
Incomplete treatment of infection	3 (6.82%)
Bad taste	3 (6.82%)
Delayed response possessing the need to switch to higher antibiotics	1 (2.27%)
Increased hospital costs	1 (2.27%)
Non-availability of 60 ml bottle	1 (2.27%)
3. A 60 ml dosage of co-amoxiclav (instead of the current 30 ml dosage) addresses the challenges of non-compliance to antibiotic therapy	
Yes	41 (85.42%)
No	7 (14.58%)
If yes, patient consideration/indication for which this dosage is advised	
Indication	
Respiratory tract infections (RTI)	32 (78.05%)
Lower respiratory tract infection (LRTI)	12 (37.5%)
Pneumonia	1 (8.33%)
Protracted bacterial bronchitis	1 (8.33%)
Upper respiratory tract infections (URTI)	15 (46.88%)
Otitis media	11 (73.33%)
Tonsilitis	2 (13.33%)
Pharyngitis	1 (6.67%)
Sinusitis	1 (6.67%)
Skin and soft tissue (SST) infections	2 (4.88%)
Severe infections	1 (2.44%)
All bacterial infections	1 (2.44%)
Patient consideration	
Weight of children	7 (17.07%)
Older children who are uncomfortable taking tablets	5 (12.2%)
Children requiring a longer duration of the course	3 (7.32%)
All children	2 (4.88%)
Children with disease progression and complications	1 (2.44%)
4. Infants born by cesarean delivery are more likely to receive probiotics in addition to antibiotics	
True	20 (41.67%)
Immature/altered gut microflora	7 (35.00%)
Deprivation of vaginal flora	5 (25.00%)
Assumption that the childbirth may have been problematic	2 (10.00%)
Dysbiosis is more in cesarean delivery	1 (5.00%)
If preterm, asphyxiated or suffering from neonatal enterocolitis	1 (5.00%)
Suppressed immunity	1 (5.00%)
Helps in antibiotic-associated diarrhea	1 (5.00%)
False	10 (20.83%)
At the time of Rx, the status of delivery mode is not known in the majority of cases	1 (10.00%)
Don't know	1 (10.00%)
Can't say	18 (37.50%)
Cesarean neonates receive probiotics depending on whether they are term or preterm babies	1 (5.56%)
Don't know	5 (27.78%)
5. Side effects profile of co-amoxiclav brands are different	
Yes	27 (56.25%)
Preparation techniques	5 (18.52%)
Quality of drug	3 (11.11%)
Strength and purity of clavulanic acid	3 (11.11%)
Pharmacokinetics and purity	1 (3.70%)
Type of cloxacillin used	1 (3.70%)
Presence of additives	1 (3.70%)
Formulation	2 (7.40%)
Depends on the molecule	1 (3.70%)
Stability of compound	1 (3.70%)
Chemical and therapeutic bioequivalence	1 (3.70%)
Brands with lactobacillus cause less diarrhea	1 (3.70%)
Experienced during clinical practice	1 (3.70%)
All are different	1 (3.70%)
No	9 (18.75%)
All are same	7 (77.78%)
Can't say	11 (25.00%)
Lack of availability of studies on side effects profile	1 (9.09%)
All are same	1 (9.09%)
6. Good manufacturing practices (GMPs) play a role in the occurrence of gastrointestinal side effects	
Yes	39 (81.25%)
Purity and quality of drug	5 (12.82%)
Purity and strength of clavulanic acid	3 (7.69%)
Deviation from best GMP practices and processes	3 (7.69%)
Sensitivity to heat and humidity	3 (7.69%)
Contamination	2 (5.13%)
Compatibility	1 (2.56%)
Better molecular mechanisms	1 (2.56%)
Gastrointestinal side effects are more common with some brands	1 (2.56%)
Variable response with different preparations and different brands	1 (2.56%)
Preparation techniques	1 (2.56%)
Particle size	1 (2.56%)
Not known	1 (2.56%)
No	3 (6.25%)
Not aware of GMPs	1 (33.33%)
Can't say	6 (12.50%)
Not aware of GMPs	3 (50%)
Individual sensitivity	1 (16.67%)
7. Frequency of prescription of probiotics with co-amoxiclav brands other than Clamp^®^	
Always	9 (18.75%)
Often	21 (43.75%)
Occasionally	17 (35.42%)
Never	1 (2.08%)

Further, 41.67% of clinicians believed that infants born by cesarean delivery are more likely to receive probiotics in addition to antibiotics. Of these, clinicians cited immature/altered gut microflora of infants (35%), deprivation of vaginal flora (25%), and an assumption that childbirth may have been problematic (10%) as the major reasons. Regarding the safety profiles, more than half of the clinicians, i.e., 56.25% agreed that the side effect profiles of co-amoxiclav brands were different. The clinicians thought that the top three reasons behind this difference were preparation techniques (18.52%), quality of the drug (11.11%), and strength and purity of clavulanic acid (11.11%). In addition, 81.25% of clinicians presumed that GMPs play a critical role in the occurrence of gastrointestinal side effects, of which 12.82% believed that purity and quality of the drug were the main reason, followed by purity and strength of clavulanic acid (7.69%), deviation from best GMP practices and processes (7.69%), and sensitivity to heat and humidity (7.69%). Amongst the various gastrointestinal side effects, diarrhea was the most reported by 95.83% of clinicians, followed by abdominal discomfort (54.17%), bloating (39.58%) and flatulence (35.42%), vomiting (20.83%), nausea (16.67%) and heartburn (6.25%).

For the responses regarding the frequency of prescription of probiotics with co-amoxiclav brands other than Clamp®, 43.75% of clinicians responded as often, while others responded as occasionally (35.42%) and always (18.75%).

Amongst the several factors considered for co-prescribing probiotics with antibiotics, a history of AAD (83.33%) was the most reported, followed by an anticipated effect on gut microbiota (70.83%), the perceived safety profile of antibiotics (33.33%), patient's age (31.25%), and recurrent RTIs (25%) (Figure 1).

Lastly, the perception of clinicians about the effectiveness and safety of Clamp® in bacterial infections among pediatric patients was assessed. More than three-fourths of clinicians (77.08%) believed that Clamp® exhibits good effectiveness against bacterial infections. Some clinicians considered Clamp® as good and the most preferred brand, which has a relatively safe side effect profile and is well-tolerated. Other reasons cited for the improved effectiveness of Clamp® include good response, good taste, lesser gastrointestinal disturbances, excellent purity, high reliability, good compliance, and easy availability (Figure 2).

Regarding the safety profile, more than two-thirds of the clinicians (70.83%) considered that Clamp® possesses a good and established safety profile. Other clinicians considered Clamp® as safe except for fewer gastrointestinal side effects (12.50%), relatively safer as compared to other brands (8.33%) and has minimal side effects (6.25%) (Figure 3).

All the formulations of co-amoxiclav in the present study were safe, as no major adverse event (AE) was observed in either of the groups. AEs reported in Group I were AAD in 1.71% (5), abdominal pain in 1.71% (5), nausea in 1.02% (3), and vomiting in 1.37% (4) patients. In contrast, in Group III, only 0.63% patient (1) complained of nausea during treatment.

## Discussion

The present study was a mixed methods research including a retrospective multicenter quantitative study and a prospective qualitative survey. The retrospective study was conducted to assess the co-prescription pattern of probiotics prescribed along with different brands of co-amoxiclav for the management of RTIs in pediatric patients using the EMR database. Further, a self-administered predefined questionnaire was conducted to evaluate the clinicians’ considerations for choosing co-amoxiclav and adding probiotics to co-amoxiclav. In the current study, the mean age of the patients was 4.05 years, and the majority were males. These findings concurred with the research reporting that acute RTIs (particularly LRTIs) frequently affect children aged less than five years [17]. Another study revealed that the incidence of RTIs is reported more in males than females [18]. Mostly, patients in the present study had URTIs compared to LRTIs. Literature has also evidenced that URTIs are the most frequent infections among children and are the primary reason for visiting pediatricians. A preschool-aged child has an average of six to 10 colds per year, and 10-15% of school-aged children have at least 12 infections per year [17].

Co-amoxiclav is a broad-spectrum antibiotic recommended by many guidelines but has reported gastrointestinal effects, particularly AAD, and is usually co-prescribed with probiotics [19,20]. The number of probiotic co-prescriptions was significantly lesser in Group I than in Groups II and III at all the visits in the current study (*p*<0.05). Further, *S. boulardii*, *B. clausii*, and LAB were the most co-prescribed probiotics in the present study. In consonance, Guo et al. (2019), in a meta-analysis including 33 studies (6352 pediatric patients), assessed the role of probiotics like *Lactobacilli spp.*, *Bifidobacterium spp.*, and *Streptococcus spp.*, or *S. boulardii *alone or in combination in preventing AAD. The incidence of AAD in the probiotic group was comparatively lesser (8%) than in the control group (19%). *Lactobacillus spp. *or *S. boulardii* (5 to 40 CFU/day) appeared to be the most appropriate probiotics for relieving AAD in children [12].

In the qualitative evaluation of clinicians' responses, five to seven days was the most recommended duration for co-amoxiclav treatment therapy. This aligns with the treatment guidelines for antimicrobial use in common syndromes issued by the Indian Council of Medical Research (ICMR) [21]. Further, non-adherence to antibiotic therapy is known to be the most crucial risk factor for treatment failure, which may lead to severe consequences that could delay recovery or deteriorate patients' health [22]. Adherence to medications in children varies widely, from 11% to 93% [23]. Goudar et al. reported that of 93 children on antibiotics, 62.36% showed poor compliance [24]. Clinicians in the present survey also agreed that non-adherence to oral antibiotic treatment poses a challenge, and antimicrobial resistance (AMR) was reported as a key consequence. Additionally, a 60 ml dosage (instead of the current 30 ml dosage) of co-amoxiclav addresses the challenges observed with non-compliance to antibiotic therapy. In consonance, in a study by Chan et al., more than 30% of participants were non-adherent to antibiotic treatment and showed a high prevalence of antibiotic resistance [25]. Moreover, non-adherence to antimicrobials also results in therapeutic failure, re-infection, and delayed response [26].

In the current survey, most clinicians reported a history of AAD and anticipated effects on gut microbiota as the crucial factors to be considered while co-prescribing probiotics with antibiotics. The responses were consistent with an online survey conducted in the seven countries of the Asia-Pacific region. Though the rates of co-prescription of probiotics varied from 16% to 39% (overall, 27%), prevention of AAD was reported as the most important factor by 62% of physicians for co-prescribing probiotics with antibiotics [11].

The clinicians in the present survey also agreed that infants born by cesarean delivery are more likely to receive probiotics with antibiotics, primarily due to immature/altered gut microflora and deprivation of vaginal flora. The process of vaginal delivery facilitates the mother's microbiome to act as the "starter culture" for the infant microbiome during birth. However, infants born by cesarean delivery have low intestinal bacterial richness and diversity. Hence, prescribing probiotics with antibiotics (when administered) may give the offspring a significant advantage in shaping eubiotic gut microbiota [13].

GMPs ensure that products are consistently produced and controlled according to quality standards. The World Health Organization (WHO) also stresses the role of GMP in combating AMR. It highlights the potential of GMPs in controlling and reducing the contamination of the environment from waste and wastewater generated from antimicrobial production [6]. Further, the various regulatory authorities also direct the production of antibiotics in segregated areas to avoid cross-contamination [27]. In concordance, in the present survey, most clinicians agreed that side effect profiles of co-amoxiclav brands are different, and GMPs play a role in gastrointestinal side effects. Diarrhea is reported as the single most common complaint after administration of co-amoxiclav, including others like nausea, vomiting, and abdominal discomfort [28].

Further, it was observed in the present study that clinicians prescribe probiotics more often with co-amoxiclav brands other than Clamp®, which might be attributed to the higher incidence of AAD with the former. Moreover, they perceived Clamp® to be effective and safe for bacterial infections in pediatric patients.

In the present study, co-amoxiclav was relatively safe in pediatric patients. The safety profile of co-amoxiclav among the pediatric population has been well established. Sugita et al. reported co-amoxiclav-related adverse events in 19% of pediatric patients suffering from bacterial sinusitis, and all reported drug-related adverse events were classified as gastrointestinal disorders [29]. Similarly, a retrospective study showed that only GI-related adverse drug reactions like diarrhea and abdominal pain were reported with the use of co-amoxiclav [30].

Strengths and limitations

Hitherto, few studies have assessed the co-prescription patterns of probiotics in pediatric patients prescribed with co-amoxiclav in Indian settings. This real-world study provides clinicians’ consideration and co-prescription patterns of probiotics prescribed with co-amoxiclav. However, certain limitations are associated with the study, such as inherent bias associated with a retrospective study design. There was the unavailability of some data parameters, such as the history of AADs, side effect profile, and comorbidities of the patients, which might have impacted the treatment response. Additionally, other than gastrointestinal tolerability, certain confounding factors could impact clinicians’ decisions while co-prescribing probiotics with co-amoxiclav.

## Conclusions

The findings of the present study demonstrated that the frequency of co-prescriptions of probiotics with Clamp® was significantly less compared to the other brands of co-amoxiclav among pediatric patients with RTIs, indicating a lower incidence of AAD with Clamp®. The safety analysis revealed that no major adverse events were observed with all the formulations of co-amoxiclav. The qualitative analysis to assess clinicians' opinions indicated that most clinicians were aware of the gastrointestinal side effects of co-amoxiclav and the benefits of probiotics in preventing them. Further, among various brands of co-amoxiclav, Clamp® was perceived as effective and safe by clinicians for managing RTIs among pediatric patients. However, larger prospective studies are required to assess the confounding factors impacting clinicians’ decisions while co-prescribing probiotics with co-amoxiclav.
